# Chemical Expansion of the Methyltransferase Reaction: Tools for DNA Labeling and Epigenome Analysis

**DOI:** 10.1021/acs.accounts.3c00471

**Published:** 2023-10-31

**Authors:** Giedrius Vilkaitis, Viktoras Masevičius, Edita Kriukienė, Saulius Klimašauskas

**Affiliations:** † Institute of Biotechnology, Life Sciences Center, Vilnius University, LT-10257 Vilnius, Lithuania; ‡ Institute of Chemistry, Department of Chemistry and Geosciences, Vilnius University, LT-03225 Vilnius, Lithuania

## Abstract

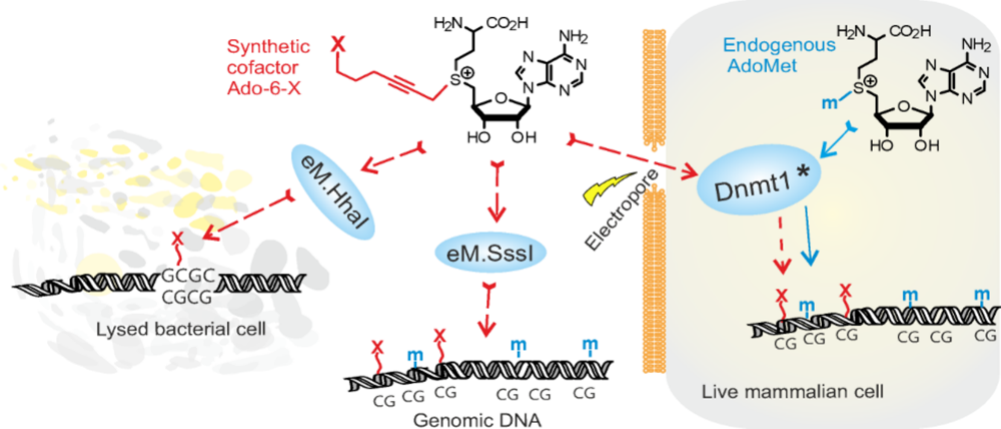

DNA is the genetic matter of life composed of four major nucleotides which can be further furnished with biologically important covalent modifications. Among the variety of enzymes involved in DNA metabolism, AdoMet-dependent methyltransferases (MTases) combine the recognition of specific sequences and covalent methylation of a target nucleotide. The naturally transferred methyl groups play important roles in biological signaling, but they are poor physical reporters and largely resistant to chemical derivatization. Therefore, an obvious strategy to unlock the practical utility of the methyltransferase reactions is to enable the transfer of “prederivatized” (extended) versions of the methyl group.

However, previous enzymatic studies of extended AdoMet analogs indicated that the transalkylation reactions are drastically impaired as the size of the carbon chain increases. In collaborative efforts, we proposed that, akin to enhanced S_N_2 reactivity of allylic and propargylic systems, addition of a π orbital next to the transferable carbon atom might confer the needed activation of the reaction. Indeed, we found that MTase-catalyzed transalkylations of DNA with cofactors containing a double or a triple C–C bond in the β position occurred in a robust and sequence-specific manner. Altogether, this breakthrough approach named mTAG (methyltransferase-directed transfer of activated groups) has proven instrumental for targeted labeling of DNA and other types of biomolecules (using appropriate MTases) including RNA and proteins.

Our further work focused on the propargylic cofactors and their reactions with DNA cytosine-5 MTases, a class of MTases common for both prokaryotes and eukaryotes. Here, we learned that the 4-X-but-2-yn-1-yl (X = polar group) cofactors suffered from a rapid loss of activity in aqueous buffers due to susceptibility of the triple bond to hydration. This problem was remedied by synthetically increasing the separation between X and the triple bond from one to three carbon units (6-X-hex-2-ynyl cofactors). To further optimize the transfer of the bulkier groups, we performed structure-guided engineering of the MTase cofactor pocket. Alanine replacements of two conserved residues conferred substantial improvements of the transalkylation activity with M.HhaI and three other engineered bacterial C5-MTases. Of particular interest were CpG-specific DNA MTases (M.SssI), which proved valuable tools for studies of mammalian methylomes and chemical probing of DNA function.

Inspired by the successful repurposing of bacterial enzymes, we turned to more complex mammalian C5-MTases (Dnmt1, Dnmt3A, and Dnmt3B) and asked if they could ultimately lead to mTAG labeling inside mammalian cells. Our efforts to engineer mouse Dnmt1 produced a variant (Dnmt1*) that enabled efficient Dnmt1-directed deposition of 6-azide-hexynyl groups on DNA in vitro. CRISPR-Cas9 editing of the corresponding codons in the genomic Dnmt1 alleles established endogenous expression of Dnmt1* in mouse embryonic stem cells. To circumvent the poor cellular uptake of AdoMet and its analogs, we elaborated their efficient internalization by electroporation, which has finally enabled selective catalysis-dependent azide tagging of natural Dnmt1 targets in live mammalian cells. The deposited chemical groups were then exploited as “click” handles for reading adjoining sequences and precise genomic mapping of the methylation sites. These findings offer unprecedented inroads into studies of DNA methylation in a wide range of eukaryotic model systems.

## KEY REFERENCES

- LukinavičiusG.; LapienėV.; StaševskijZ.; DalhoffC.; WeinholdE.; KlimašauskasS.Targeted labeling of DNA by methyltransferase-directed transfer of activated groups (mTAG). J. Am. Chem. Soc.2007, 129, 2758–2759.1730926510.1021/ja0691876(1) The first demonstration of the utility of the mTAG approach for sequence-specific derivatization and labeling of DNA.
- LukinavičiusG.; TomkuvienėM.; MasevičiusV.; KlimašauskasS.Enhanced Chemical Stability of AdoMet Analogues for Improved Methyltransferase-Directed Labeling of DNA. ACS Chem. Biol.2013, 8, 1134–1139.2355773110.1021/cb300669x(2) Establishment of key design principles underlying the chemical stability and transalkylation activity of propargylic AdoMet cofactor analogs.
- KriukienėE.; LabrieV.; KhareT.; Urbanavičiu̅tėG.; LapinaitėA.; KoncevičiusK.; LiD. F.; WangT.; PaiS.; PtakC.; GordevičiusJ.; WangS. C.; PetronisA.; KlimašauskasS.DNA unmethylome profiling by covalent capture of CpG sites. Nat. Commun.2013, 4, 3190.10.1038/ncomms319023877302(3) The first implementation of the mTAG approach as an analytical tool to query the methylation status of CpG-sites in mammalian genomes and to determine the cell-type-specific genome-scale “unmethylome” profiles.
- StankevičiusV.; GibasP.; MasiulionytėB.; GasiulėL.; MasevičiusV.; KlimašauskasS.; VilkaitisG.: Selective chemical tracking of Dnmt1 catalytic activity in live cells. Mol. Cell2022, 82, 1053–1065.3524544910.1016/j.molcel.2022.02.008PMC8901439(4) The first demonstration of mTAG labeling of DNA in vivo enabling selective covalent tagging and precise genomic mapping of epigenetic marks deposited by an individual DNMT methyltransferase enzyme in live mammalian cells.

## 1 Introduction

**Figure 1:**
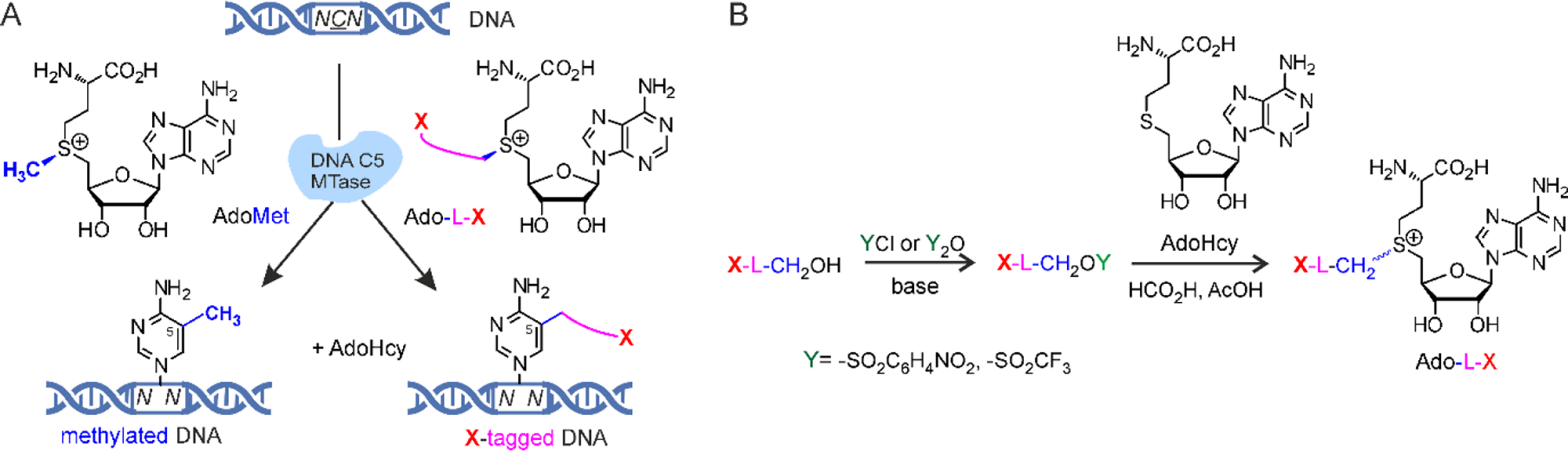
(A) Methyltransferase-directed sequence-specific transfer of a methyl group onto the fifth position of the target cytosine residue (underlined) in DNA from AdoMet (biological methylation) or transfer of an extended moiety carrying a linker L and functional group X from a synthetic cofactor analog Ado-L-X (targeted derivatization). (B) General approach for chemical synthesis of extended cofactor analogs Ado-L-X by *S*-alkylation of AdoHcy, under acidic conditions, with corresponding 4-nitrobenzenesulfonates or trifluoromethanesulfonates obtained from corresponding alcohols X-L-CH_2_OH.

The genetic book of life is encrypted in long linear DNA strands consisting of four major types of coding units. Besides these major nucleotides A, C, G, and T, smaller amounts of a fifth base, 5-methylcytosine (m^5^C, originally named epi-cytosine), were identified in animal DNA back in 1948.^5^ This minor base as well as all other methylated nucleotides in DNA is produced via enzymatic modification of one of the major nucleobases, cytosine, by enzymes called methyltransferases (MTases). These enzymes catalyze the transfer of methyl groups from the ubiquitous cofactor *S*-adenosyl-l-methionine (AdoMet or SAM) to their biological target on DNA (Figure 1A, left). In vertebrate DNA, the m^5^C residues are largely confined to CG dinucleotides (28 million in the human genome), but their distribution in the genome is highly variable across different genetic loci, cells, and organisms and is dependent on tissue, age, sex, diet, and disease. m^5^C is a key epigenetic mark involved in coordinated regulation of tens of thousands genes in a myriad of cell-type-specific programs during development, functioning, and interactions with the environment of multicellular organisms.

Besides m^5^C, microbial DNAs have been found to contain *N*6-methyladenine^6^ and then later *N*4-methylcytosine.^7^ In prokaryotes and archaea, all three classes of DNA methylation occur sequence specifically, and thousands of distinct recognition sequences (REBASE, http://rebase.neb.com) have been identified or inferred based on their DNA modification profiles.^8^ Distinct combinations of such sequence-specific methylation profiles make a species-specific marking of host DNA that uniquely distinguishes it from invading foreign DNA. In addition to their relatively compact size, the inherent integration of sequence-specific recognition with covalent modification made prokaryotic MTases attractive models for fundamental studies of DNA–protein interactions. Among the three classes, m^5^C-specific MTases (C5-MTases) are the most conserved family of proteins and distinguish themselves in that their catalytic reaction involves covalent activation of the target cytosine.^9^ For more than a decade, our favorite model system for detailed mechanistic studies of DNA methylation and DNA base flipping was a GCGC-specific MTase, M.HhaI.^10−14^

After gaining in-depth mechanistic insight, we turned to directed engineering of these enzymes because the feeling was that the best proof of really understanding something is the ability to modify it in a predictable manner. The same mentioned features of bacterial DNA MTases (compact size, sequence recognition, and covalent catalysis) made them also attractive candidates for engineering DNA labeling tools. Since the transferred methyl groups are poor reporters and not readily amenable to further derivatization, one strategy to unlock the biotechnological power of these enzymes is to make them transfer “prederivatized” versions of the methyl group from synthetically designed AdoMet analogs.

## 2 mTAG: Methyltransferase-Directed Transfer of Extended Groups from Synthetic Cofactors

AdoMet, originally described as the “ATP-activated form of methionine”^15^ is the major methyl group donor and the second most ubiquitous cofactor after ATP in all living organisms. Although almost any part of the AdoMet molecule can be utilized,^16^ biological transmethylation is the prevalent role of AdoMet. The positively charged sulfonium center induces a partial electron deficiency on the adjoining methyl group facilitating S_N_2 transfer reactions^17^ onto biological nucleophiles.

**Figure 2:**
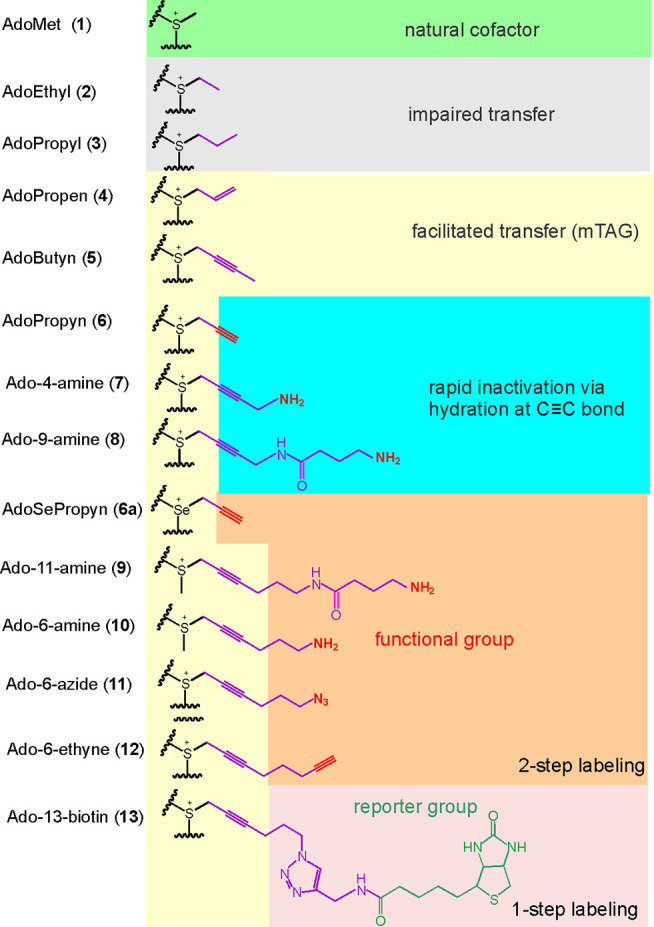
Structural and functional comparison of AdoMet and its extended synthetic analogs related to this work.

The idea to functionalize the sulfonium-bound methyl group in AdoMet by replacing it with a linear carbon chain containing a desired functional or reporter group seemed like a straightforward strategy. However, early attempts to “extend” the methyl group proved quite discouraging as the transalkylation rates decreased dramatically upon addition of just two carbon atoms.^18^ The observed decline echoes with the rates of chemical S_N_2 reactions (methyl ≫ ethyl > propyl), which manifest both steric and electronic effects of the bulkier and electron-donating −CH_2_– group replacing an H atom (Figure 2). Therefore, further engineering of the methyltransferase reaction by installing even longer functionalized groups offered poor perspectives for practical applications. On the other hand, faster S_N_ reactions are known to occur with allylic, propargylic, and benzylic systems (see Table 4.1 in ref (19)) whereby a π orbital of the adjoining unsaturated bond can provide conjugative stabilization of an sp^2^ transition state formed on the transferable carbon. In a collaborative effort with the group of Elmar Weinhold (RWTH Aachen, Germany), we proposed that similar “re-activation” of the extended side chain could be achieved by placing an sp^2^ or sp^1^ carbon next to the transferable carbon atom (β position to the sulfonium center). Using all three types of DNA MTases including our favorite M.HhaI, we indeed demonstrated that the MTase-catalyzed transalkylations of DNA with synthetic cofactors carrying allyl and but-2-ynyl groups were much more efficient as compared to the saturated *n*-propyl group (butynyl > allyl ≫ ethyl > propyl).^20^

The transalkylations occurred in a sequence- and base-specific manner with turnover rates in the minute time scale, indicating that such targeted derivatization of DNA could potentially be adapted for routine laboratory use. The allylic and propargylic series were termed doubly activated cofactors,^21^ and the whole chemo-enzymatic approach was named mTAG (methyltransferase-directed transfer of activated groups).^1^

**Figure 3:**
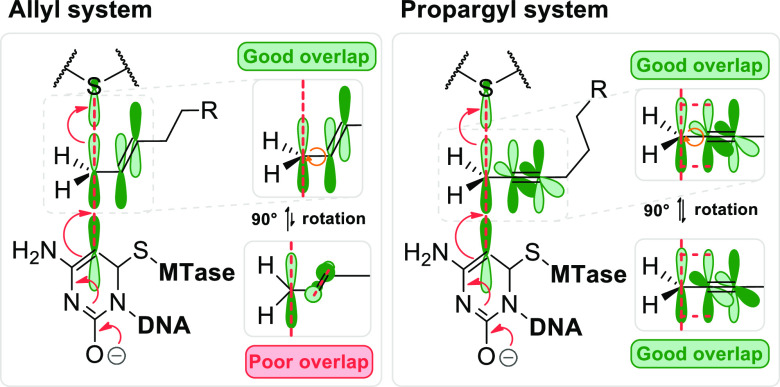
Proposed mechanism for the facilitated transfer of an extended sulfonium-bound allyl (left) and propargylic (right) side chain by a DNA cytosine-5 MTase via π-orbital conjugation (green) of the adjacent unsaturated carbon with the sp^2^-like transition state. These interactions preclude nonplanar conformations of the allylic (but not propargylic) side chain which may limit a steric compatibility of the cofactor within the active site of a directing MTase.

Here, we chose to focus on the propargylic cofactors mainly due to the following two reasons. One theoretical consideration was that sp^2^ systems (allylic, benzylic) can conjugate (i.e., spatially align with) the transition state p orbital only in two possible conformations of the **C**–C bond when the **C**–C=C plane is perpendicular to the direction of attack/p orbital (Figure 3). No such conformational restrictions exist for the propargylic systems since the π orbitals at the sp^1^ carbon are independent of the **C**–C bond rotation (unless the triple bond is conjugated with other unsaturated systems in the side chain). It is also known that propargylic systems are somewhat more reactive electrophiles than allylic ones in S_N_ reactions. Benzylic groups seemed too bulky to be the first choice for the enzyme-catalyzed reactions. For these reasons or other, it turned out that indeed the C5-MTases were much more active with sp^1^ compounds (see below), although other classes of MTases showed none or even the opposite cofactor preferences.

To take the mTAG strategy further one needed a general synthetic approach for production of AdoMet analogs with diverse extended groups. De la Haba et al. described regioselective *S*-methylation of AdoHcy to AdoMet under acidic conditions,^22^ which render transient protection of the *N*-nucleophilic positions in the molecule. We adapted this approach for “direct charging” of AdoHcy with the activated carbon chains by using appropriate alkylating agents (Figure 1B). Simple AdoMet analogs were obtained using commercially available 3-bromo-1-propene or triflate-activated but-2-yn-1-ol.^23^ However, with propargylic side chains carrying heteroatoms/functional groups, halogenides did not offer good conversions, whereas triflates often led to undesired side reactions. Here, we turned to arylsulfonates, which permitted fine tuning of the reactivity by selecting proper substituents in the aryl moiety. The best results were achieved with 4-nitrobenzenesulfonates (nosylates), which can be readily obtained from the corresponding alcohols (Figure 1B).^2,24^ In most cases, the *O*-nosylated side chains proved stable enough to be isolated and stored until needed and were sufficiently active to give nearly full conversions in overnight reactions. Under these conditions, *N*-Boc protection was required for terminal amine, whereas no protection was required for azide or alkyne. Cofactors with large reporter groups such as chromophores or biotin were obtained by further appending the functionalized cofactors via suitable conjugation chemistries under mild acidic conditions.^25−29^ This general route afforded multimilligram amounts of cofactor analogs, as diastereomeric mixtures.^2,24,30^

## 3 Improved Cofactors for MTases

**Figure 4:**
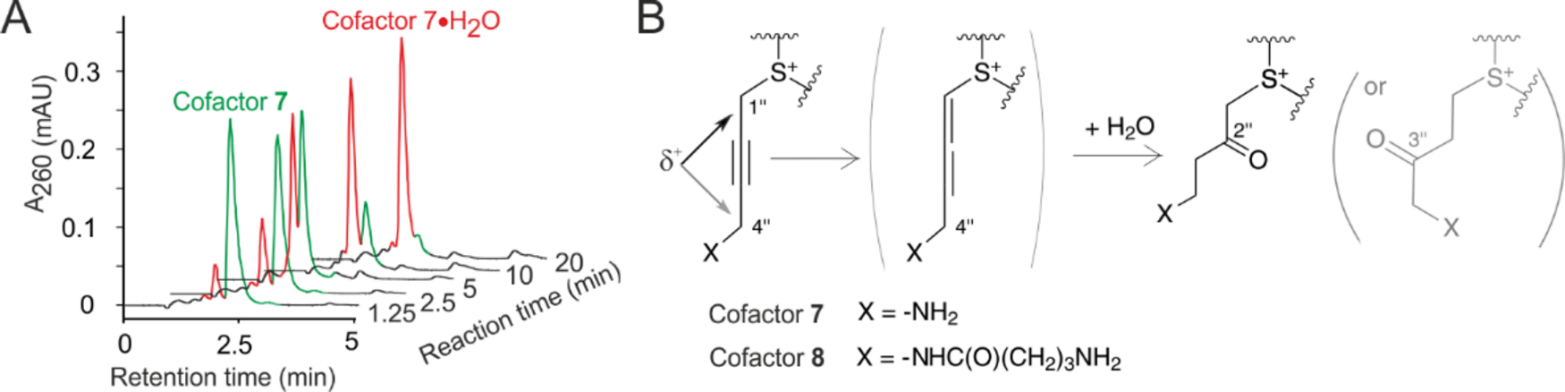
Rapid inactivation of 4-substituted propargylic AdoMet analogues in aqueous buffers. (A) Time-course HPLC chromatograms of reaction products obtained with cofactor **7** in M.HhaI buffer (pH 7.4) at 37 °C. (B) Proposed mechanism of hydrolytic inactivation of 4-substituted propargylic AdoMet analogues. Adapted with permission ref (2). Copyright 2013 American Chemical Society.

Using the above approach, our group pioneered the design and numerous applications of cofactors with extended propargylic side chains. However, despite success in certain applications,^1^ we found that the simplest propargylic cofactors containing a one-carbon linker and a functional group (4-X-but-2-yn-1-yl series, X = −NH_2_ or −NHCO–(CH_2_)_3_–NH_2_) suffered from a rapid loss of activity under physiological conditions.^2^ Analysis of the inactivation products showed that a water molecule is added to the side chain of the cofactor in a pH-dependent manner (Figure 4A). Since no such reactivity was observed with the aliphatic side chains (but- and pent-2-yn-1-yl) under similar conditions, we concluded that the presence of an electronegative group (protonated amine or amide) in proximity to the sulfonium-activated triple bond increases the electron deficiency on C4 (which is manifested by changes of the ^1^H NMR chemical shifts at H4′′ from ∼2 to 4 ppm) and the propargyl moiety. Altogether, we presumed that the latter group rearranges into an allenic system followed by fast hydration to an inactive but-2-oxo derivative (Figure 4B). A similar mechanism has been proposed for the rapid hydration of the AdoMet analog carrying an unsubstituted sulfonium-bound propargyl group, which was resolved by replacing the S atom in the onium center with Se (cofactor **6** and **6a**).^31^

To verify this hypothesis (and alleviate the stability problem), we synthetically increased the separation between an electronegative group and the triple bond from one to three carbon units. To our satisfaction, the 6-X-hex-2-ynyl cofactor series with terminal amino, azide, or alkyne groups (**9**–**13**) showed a dramatically improved stability (*t*_1/2_ > 3 h at physiological conditions) and transalkylation activity.^2^ These new cofactors proved highly useful for two-step sequence-specific labeling of DNA and other biopolymers worldwide.

In our and other groups worldwide, a series of mTAG cofactors with diverse side chains was synthesized and studied for derivatization and labeling of DNA, RNA, and protein targets.^31−34^ Cofactors with shorter side chains (3–5 carbon units) were typically intended for probing wild-type MTases and interrogation of a wide spectrum of cellular enzymes and even entire methylomes in cells or cell extracts. However, our selection of a longer, hex-2-ynyl (−CH_2_–C≡C–(CH_2_)_3_–X), moiety as the basic transfer unit was motivated by the generally poor acceptance of bulky groups by many wild-type MTases. This is a particularly important feature for confining the transalkylation activity to the engineered MTase in the context of a vast variety of endogenous AdoMet-dependent MTases present in cells.

## 4 Engineering Bacterial DNA C5-MTases and Their In Vitro Applications To Study Mammalian Methylation

**Figure 5:**
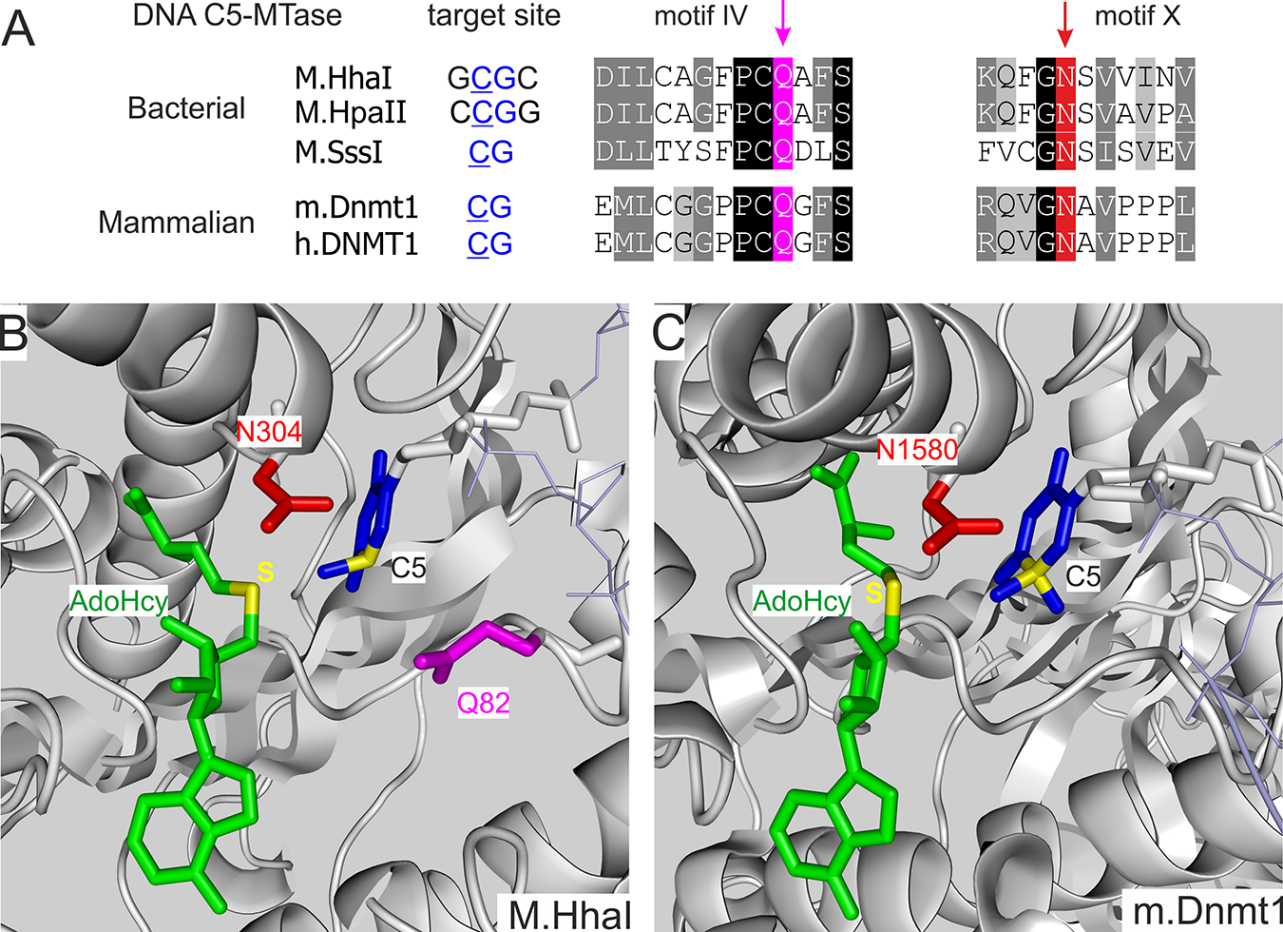
Structure-based engineering of DNA C5-MTases for acceptance of extended AdoMet analogs. (A) Sequence alignment of CG-specific DNA C5-MTases at conserved motifs IV and X. Arrows point at active-site residues subjected to alanine replacements. (B and C) Crystallographic models around the bound cofactor (AdoHcy, green) and the flipped out target cytosine residue (blue) show positions of the engineered residues in M.HhaI (PDB ID: 6MHT) and m.Dnmt1 (PDB ID: 6W8W).

During these studies we also learned that bacterial DNA C5-MTases were poorly active with cofactors that carry side chains longer than four carbon units, pointing at steric constrains imposed by the catalytic center of these enzymes. We therefore performed structure-guided engineering of the cofactor pocket of the M.HhaI MTase by shortening nonessential residues that potentially were in steric conflict with the extended side chain of a modeled cofactor analog.^35^ Two of the three identified residues (Q82 and N304) occur in conserved sequence motifs IV and X which are shared by all C5-MTases (Figure 5A and 5B). Our experiments showed that the Q82A mutant displayed a small enhancement of the transalkylation rate but led to considerable reduction of the methylation rate. In contrast, the N304A mutation was essential for the acceptance of bulky payloads such as Ado-11-amine (cofactor **9**, Figure 2). The double replacement (Q82A/N304A) conferred a substantial improvement of the transalkylation activity and a modest reduction of the methyltransferase activity with full retention of the sequence specificity. Notably, biochemical studies of a representative variant indicated that the described mutations lead to enhanced catalytic rates rather than improved cofactor binding. Structural considerations suggested that a broader channel in the cofactor pocket permits a more favorable precatalytic conformation for an extended side chain but leads to weaker binding and a less favorable conformation of the methyl group of AdoMet. The observed switch in cofactor selectivity permitted efficient M.HhaI-directed mTAG labeling with a large variety of functional or reporter groups even in the presence of the natural cofactor AdoMet.^35^

Similar effects were confirmed to hold for other 3 out of 3 examined bacterial C5-MTases that we sterically engineered at the identified conserved positions in the absence of crystal structures.^35^ Of particular interest were the CpG-specific MTases M.SssI (variant Q142A/N370A, Figure 5A)^3^ and M.MpeI (Q136A/N347A, unpublished data and refs (28) and (36)), which can be targeted to modify the methylation sites in mammalian genomes. Altogether, owing to the improved cofactor acceptance by directing MTases and prolonged cofactor lifetimes, the mTAG technology has since been used by us and many groups worldwide for applications spanning from DNA single-molecule genotyping to high-resolution studies of whole methylomes in mammalian organisms.^37−42^

In a proof of principle study, a two-step M.HhaI-directed mTAG labeling was employed to attach fluorophores on 215 GCGC sites in bacteriophage lambda DNA (48.5 kb).^37^ The DNA molecules were then stretched using an evaporating droplet technique, and the physical positions of the fluorophores along individual DNA strands were recorded at subdiffraction resolution (10 nm or 20 bp) using dSTORM imaging. The spatial distribution of the labeled GCGC sites (termed “fluorocode”) provided a characteristic machine-readable representation of the lambda DNA sequence akin to a conventional barcode. The fluorocode concept has been taken further by our collaborators,^43^ other groups,^44^ and independently by the company Bionano for submegabase optical genotyping of large genomes.^45^

**Figure 6:**
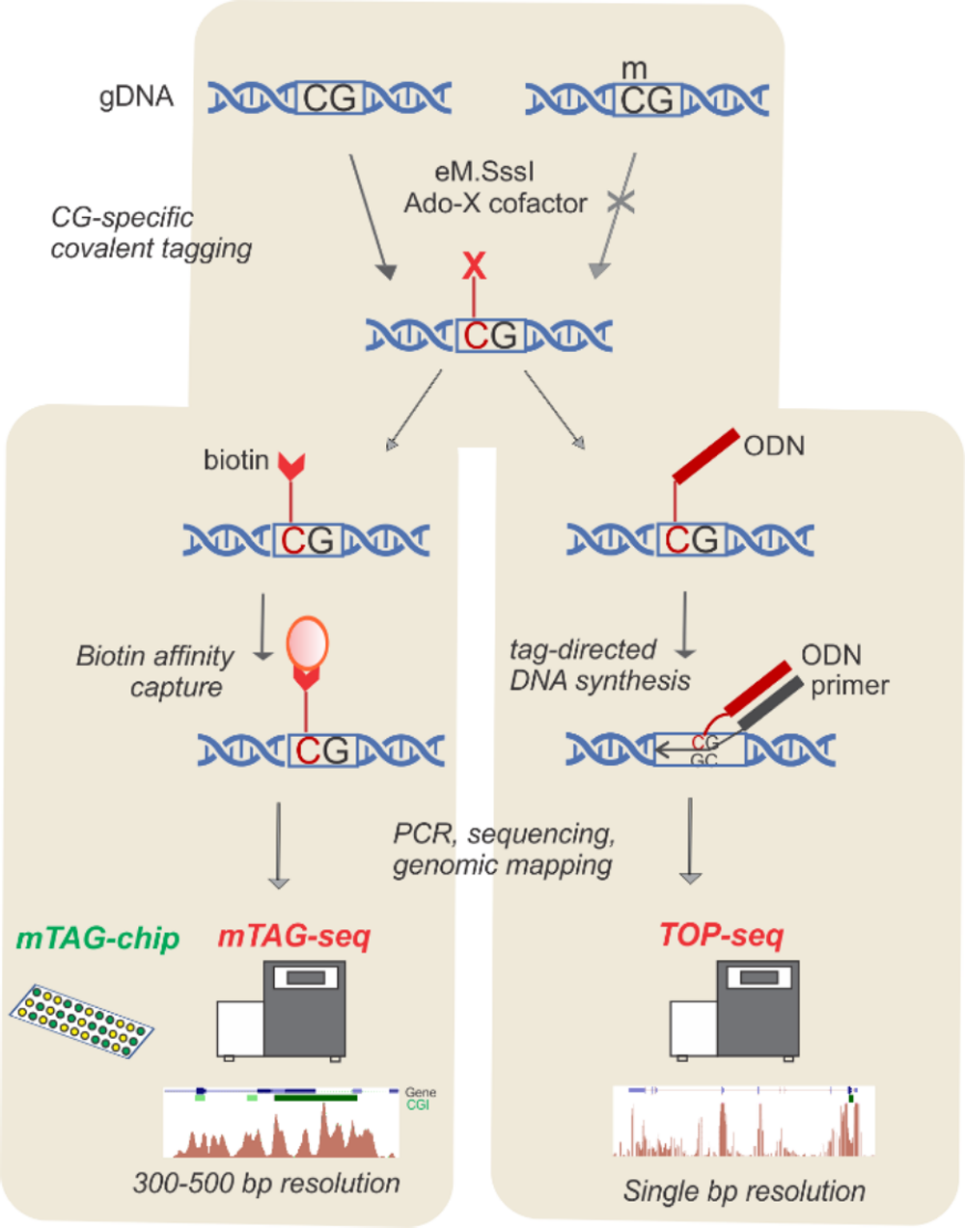
Application of mTAG labeling for whole genome profiling unmodified CG sites (unmethylome) in mammalian DNA. Schematic outline of the workflows of the mTAG-seq (left) and TOP-seq (right) approaches for whole genome profiling of the methylation status of CG sites in mammalian DNA.

Profiling the modification status of tens of millions of CG sites in the genome is a challenging task, and numerous epigenomic techniques have been developed that differ in their throughput, sensitivity, resolution, and cost. Our key concept for advancing epigenome profiling was using MTase-directed labeling for covalent tagging of the unmodified fraction of CG sites in the genome, termed “unmethylome”. Since inherently methylated CG sites remain untagged, this gives an inverse but equally informative view of the methylation status of the CG targets in the genome. In the first study of CG methylation in mammalian genome we used a two-step covalent biotin labeling directed by the engineered variant of M.SssI.^3^ The enriched biotin-labeled DNA fragments were amplified and analyzed on DNA microarrays (mTAG-chip) or by next-generation sequencing (mTAG-seq) to permit their mapping onto a reference genome at a resolution of 200–500 bp (defined by the length of amplifiable DNA fragments, Figure 6, left).

A further advance in resolution down to a single CG site was achieved by chemical tethering of a DNA oligonucleotide (instead of biotin) to the azide-derivatized unmodified genomic CG sites (Figure 6, right). The tethered oligonucleotide facilitated nonhomologous priming and strand extension by the DNA polymerase at the attachment site. This newly discovered priming reaction (named tethered oligonucleotide-primed sequencing, TOP-seq) afforded direct read out of the adjoining regions and thus precise mapping of the methylation sites in the genome.^46^ Owing to the robust and nondestructive nature of the labeling procedure, the generated TOP-seq maps of unmethylated CG sites proved instrumental for discerning subtle tissue-specific methylation differences on a local or whole-genome scale.^26,46^ For example, an adaptation of the TOP-seq protocol for karyotyping of cell-free DNA circulating in maternal blood enabled detection of fetal trisomy of chromosome 21 from miniscule amounts of sample.^47^

## 5 Engineering Mammalian Cells for Chemical Tracking of Dnmt1 Catalysis In Vivo

**Figure 7:**
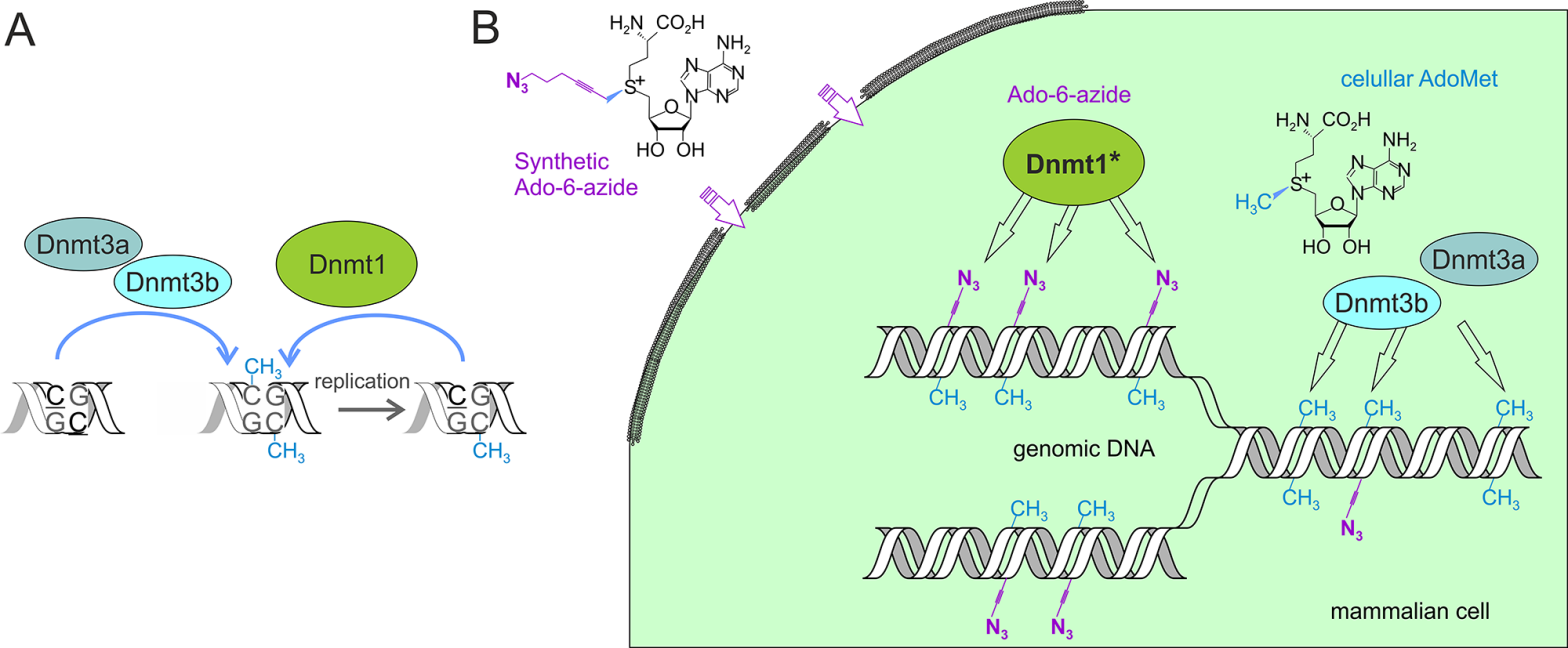
(A) Interplay of the Dnmt1, Dnmt3a, and Dnmt3b catalytic activities in establishing and maintaining the cytosine-5 methylation patterns of genomic CG sites in mammalian DNA. (B) Chemical tracking of Dnmt1 activity in vivo by pulse internalization of synthetic Ado-6-azide cofactor (**11**) into Dnmt1*-engineered mammalian cells.

Methylation of cytosine to m^5^C is the prevalent covalent epigenetic mechanism in higher eukaryotes. DNA methylation in mammals is brought about by three independently regulated DNA methyltransferases (DNMT1, DNMT3A, and DNMT3B).^48,49^ The first characterized mammalian methylase, DNMT1, preferentially acts on hemimethylated CpG sites^50^ and is mainly responsible for maintaining pre-existing methylation patterns after DNA replication. The other two major types of mammalian methylases, DNMT3A and DNMT3B, show no such substrate preference and are assigned major roles in methylation of unmodified genomic regions (de novo methylation) (see Figure 7A). Loss of the DNMT1 function is directly linked to tumorigenesis and chromosomal instability,^51,52^ whereas mutations in the DNMT3B gene cause a severe autosomal disease, called ICF syndrome.^53−55^ Disruption of each individual DNMT gene in experimental mice leads to a distinct but eventually lethal phenotype, emphasizing the complexity and importance of DNA methylation in mammalian development.

All three mammalian DNMTs contain a catalytic domain, located in the C-terminal part, and a large multidomain N-terminal part, which varies both in size and in structure.^49^ The smaller C-terminal part, which is conserved between eukaryotic and prokaryotic MTases, is responsible for cofactor binding and catalysis.^9^ The N-terminal part mediates interactions of the enzymes with other proteins, DNA, and chromatin and thus serves to target them to their nuclear localizations. Crystal structures of mammalian DNMT1 and DNMT3A show a nearly full overlap of the protein backbone atoms and active-site residues among themselves and M.HhaI further,^10,56−58^ confirming a high structural conservation of the catalytic center of C5-MTases.

Inspired by the successful engineering of bacterial MTases for the transalkylation reactions,^35^ we asked the question of whether substantially more complex mammalian methyltransferases could be similarly engineered, ultimately leading to the implementation of mTAG labeling inside mammalian cells. That would enable one to selectively track the genuine catalytic action of an individual DNMT enzyme during cell reprograming and other key developmental events—something that has not been achieved before. In a similar manner, we used structure-guided engineering of the mouse DNMT1 (Figure 5) to enable the transfer of the 6-carbon linear moieties containing a functional azide group onto DNA from corresponding cofactor analogs. These experiments produced a DNMT1 mutant in which a single mutation (N1580A corresponding to N304A in M.HhaI and N370A in M.SssI) conferred a 8400-fold improvement in cofactor selectivity (Ado-6-azide vs AdoMet) as compared to the WT enzyme! Importantly, we found that the engineered Dnmt1 retained partial methylation activity and was capable of transferring extended groups in the presence of competing AdoMet in vitro.^4^

To establish endogenous expression of the engineered version of the enzyme in mouse embryonic stem cells, we installed the corresponding codon in the DNMT1 alleles using CRISPR-Cas9 genome editing. Since AdoMet and its analogs show poor cell permeability, the remaining major obstacle was to figure out a mild way for bringing the desired cofactor inside the mammalian cells. Metabolic in-cell production using the corresponding methionine derivatives has previously been described for AdoMet analogs with short transferable groups (3 carbon atoms).^59,60^ This approach often requires methionine deprivation, leading to dramatically altered DNA methylation and cell phenotypes.^61,62^ To avoid these limitations, we chose to examine if temporary generation of membrane pores by electroporation, which has been immensely instrumental for bringing foreign genetic matter into diverse types of cells,^63^ might work in this case too. After extensive experimental trials, we elaborated experimental conditions that permitted well-reproducible pulse labeling of genomic DNA by exogenous Ado-6-azide in the knock-in mouse cells but showed no discernible effects on the functionality and viability of the ESCs. The internalized Ado-6-azide cofactor is selectively utilized by the engineered Dnmt1 to tag its genuine methylation sites, whereas in its absence, the enzyme performs normal methylation functions using endogenous AdoMet. Intragenic incorporation of the azide tags was dose and time dependent, and the attained tagging levels in pulse labeling experiments were around 1% of endogenous m^5^C.^4^ As the genomic DNA is methylated to its natural levels before Ado-6-azide entry, the chemical labeling reports on DNMT1 methylation events at newly emerging target sites that become available upon execution of epigenetic programs in proliferating or differentiating cells during the labeling time window (1–6 h). By fine tuning certain experimental variables (cofactor concentration, pulse duration, genome copy number), the system can be tailored to meet a range of experimental demands.

Genomic mapping of the tagged sites was based on exploiting the azide “click” handles for reading adjoining sequences using the above-described TOP-seq technique.^46^ The generated Dnmt-TOP-seq maps permitted comprehensive high-resolution analysis of individual enzyme-specific methylation landscapes in mouse ESCs. These maps showed good general agreement with local and genome-wide features obtained by the gold-standard whole genome bisulfite sequencing.

## 6 Summary and Outlook

This Account describes the development of an enabling technology from a proof-of-principle demonstration to a variety of applications involving targeted covalent derivatization and analysis of DNA^64^ (as well as RNA, proteins, and small molecules)^34,65,66^ by numerous laboratories worldwide. Ultimately, we propose the first general approach that permits high-resolution genome-wide “tracking” of methylation events carried out by an individual Dnmt enzyme in live mammalian cells. Current studies are aimed at exploiting this approach for selective tracking of Dnmt1 action during differentiation of pluripotent cells to precursor or somatic lineages. Due to the particularly high homology of the catalytic motifs of the eukaryotic DNMT proteins, the established approach should in principle be applicable for studies of human and other vertebrate cells and organisms. Moreover, the acceptance of bulky extended cofactors such as Ado-13-biotin (**13**) by the engineered Dnmt1^4^ offers immense flexibility in tracking modalities. For example, certain deposited chemical tags should be readily discernible by single-molecule DNA sequencing technologies such as Oxford Nanopore (Tomkuvienė, M.; Balčiu̅nas, J.; . Klimašauskas, S. Unpublished observations) and PacBio SMRT,^67,68^ or appended fluorescent tags could be exploited for 3D genomic mapping using super-resolution imaging technologies. The availability of a new type of epigenomic information (Dnmt-selective methylation profiles) will facilitate the resolution of many puzzles of how genomic methylation is established and maintained during development, senescence, and disease. In another vein, we found that the deposited tags can render nucleosome repositioning in DNA,^69^ which opens new avenues in manipulating epigenetic processes in live cells.
